# Ravages of Scurvy in Russia

**Published:** 1852-02

**Authors:** 


*Ravages of Scurvy in Russia.*—In the new Russian provinces of Bessarabia, &c., an epidemic of scorbutus broke out in December, 1848, continued to spread and become more serious till May, 1849, and in June began to subside gradually. The cause was by universal consent referred to the scarcity of means of subsistence, consequent upon a short grain crop, a failure among the garden vegetables, a severe winter, and deficient supply of fuel. The German colonists, whose circumstances were more comfortable than those of the rest of the population, escaped.—Districts in which horse-flesh or fish were used as food, suffered comparatively little, yet the epidemic seemed to be guided in its spread to certain localities, where famine was not felt, by miasmatic influence. The symptoms were those usually described: In the severer cases, general weakness, indurations of the muscles, especially those of the inferior extremities, hemorrhages, &c.; as sequelæ, dropsies, dysentery, passive inflammations of internal organs, gangrene, and typhus of a putrid type were observed. The means of cure found most efficacious were, acetate of iron, with bitters and aromatics, milk diet, yeast, turpentine, both internally and externally, tar-water, and creasote. The total number of scorbutic cases amounted to 260,444, of which 67,958, or more than a fourth part, proved fatal.—*Monthly Jour. Med. Scien.*, Nov. 1851, from *Schmidt's Jabrbücher*.

